# Aortitis and uveitis. A challenging case of Takayasu or Behcet disease?

**DOI:** 10.1186/1546-0096-12-S1-P354

**Published:** 2014-09-17

**Authors:** Giancarla Di Landro, Federica Cuoco, Sofia Torreggiani, Maria Maddalena D'Errico, Giovanni Filocamo, Fabrizia Corona

**Affiliations:** 1 UOS Reumatologia Pediatrica, Pediatria a Media Intensità di Cura, Fondazione IRCCS Cà Granda, Ospedale Maggiore Policlinico, Milan, Italy

## Introduction

Childhood vasculitis is a group of conditions that are defined as the presence of blood vessel inflammation, and they are grouped in base of the size of vessels involved. In 2008 were presented the PRES revised classification criteria for childhood vasculitis.

## Objectives

We describe the case of a vasculitis interesting the aortic arch in a boy with panuveitis, HLA B51 positive and proteinuria.

## Methods

We presented a challenging case of vasculitis classified considering the EULAR/PRINTO/PRES c-Takayasu Arteritis criteria of 2008 and the International Criteria for Behcet disease of 2013 (ICBD)

## Results

B. is a ten years old boy born in Columbia. At the age of nine, he presented fever that lasted for about 1 month. In the suspect of Kawasaki disease, he was administered Immunoglobulin, without resolution, that persisted until administration of intravenous steroid. His clinical conditions were characterized by asthenia, arthralgia and photophobia. He also presented a heart murmur of 2/6 at centrum cordis. He presented high inflammatory markers, HLA-B51 positivity and proteinuria. Chest radiography, abdomen ultrasound, brain MRI, lumbar puncture and cardiac ultrasound were negative. Instead total body PET and MRI showed inflammation of aortic arch and signs of previous pericarditis. The eye examination showed panuveitis with retinitis. The therapy administered was based on sistemic and ocular steroids and on mycophenolate mofetil with benefit. Table 1.

**Table 1:** Clinical features of our patient and differential diagnosis based on PRES c-TA and BD criteria

EULAR/PRINTO/PRES c-TA criteria	Our patient	ICBD-point score system *Sign/symptom*	Score	Our patient
Angiographic abnormality	**Yes**	Ocular lesion	**2**	**Yes**

1. Pulse deficit or claudication	Not	Genital aphthosis	2	Not

2. Blood pressure discrepancy	Not	Oral aphthosis	2	Not

3. Bruits	Not	Skin lesions	1	Doubtful

4. Hypertension	Not	Neurological manifestations	1	Not

5. Acute phase reactant	**Yes**	Vascular manifestation	**1**	**yes**

		Positive pathergy test	1	not performed

## Conclusion

Making the diagnosis of vasculitis is often challenging, because presenting symptoms may be subacute, non-specific and non diagnostic. Our patient had clinical manifestations, signs and symptoms of TA and BD. The TA criteria were satisfied by the presence of the aorta thickening and high inflammatory markers, however clinical features such as pericarditis, uveitis and HLA-B51 positivity are suggestive for BD, even if the BD criteria are not fulfilled. Vasculitis classifications are useful for patient categorization however often other clinical characteristics must be considered in distinguishing ambiguous situations.

## Disclosure of interest

None declared

